# Evaluating the effectiveness and safety of acupuncture on serum uric acid in asymptomatic hyperuricemia population: a randomized controlled clinical trial study protocol

**DOI:** 10.3389/fendo.2023.1218546

**Published:** 2023-10-13

**Authors:** Ling-ling Yu, Chen-nan Li, Meng-yue Fang, Yan Ma, Bo Wang, Feng-ping Lin, Wen-hua Liu, Sheng-hao Tu, Zhe Chen, Wen-xi Xie, Rui-yuan Zhang, Yao Huang, Cui-hong Zheng, Yu Wang

**Affiliations:** ^1^ Institute of Integrated Traditional Chinese and Western Medicine, Tongji Hospital, Tongji Medical College, Huazhong University of Science and Technology, Wuhan, Hubei, China; ^2^ The Second School of Clinical Medicine, Tongji Medical College, Huazhong University of Science and Technology, Wuhan, Hubei, China; ^3^ Department of Rehabilitation Medicine, Wuhan No.1 Hospital, Tongji Medical College, Huazhong University of Science and Technology, Wuhan, Hubei, China; ^4^ Department of Endocrinology, Xianning Central Hospital, Hubei University of Science and Technology, Xianning, Hubei, China; ^5^ Clinical Research Center, Tongji Hospital, Tongji Medical College, Huazhong University of Science and Technology, Wuhan, Hubei, China

**Keywords:** manual acupuncture, sham acupuncture, asymptomatic hyperuricemia, serum uric acid, randomized controlled trial, protocol

## Abstract

**Background:**

The clinical dangers of asymptomatic hyperuricemia to human health have become increasingly prominent over the past 20 years. Previous studies have shown the potential benefits of acupuncture on uric acid levels in the body. However, definitive evidence is lacking. Our objective is to evaluate the efficacy and safety of acupuncture on serum uric acid (SUA) in individuals with asymptomatic hyperuricemia.

**Methods:**

This is a randomized, single-blind, sham-controlled trial. A total of 180 eligible patients with asymptomatic hyperuricemia will be recruited at three hospitals in China. Patients will be randomly assigned in a 1:1 ratio to receive 16 sessions of manual acupuncture or sham acupuncture for 8 weeks. Patients will be followed up for 12 weeks. The primary outcome will be the change in SUA levels at week 8 after randomization. Secondary outcomes will include dynamic changes in SUA levels, efficacy rates, proportion of gout flare, body weight, and acute medication intake. The MGH Acupuncture Sensation Scale and adverse events related to acupuncture will be measured after each treatment. A blinding assessment will be performed on patients who receive at least one session of acupuncture. Data analyses will be performed on a full analysis set and a per-protocol set.

**Ethics and dissemination:**

Ethics approval has been obtained from the Clinical Trial Ethics Committee of Tongji Medical College, Huazhong University of Science and Technology (approval no. 2021-S135). Written informed consent will be obtained from enrolled patients. The findings will be disseminated in a peer-reviewed journal.

**Clinical trial registration:**

ClinicalTrials.gov identifier, NCT05406830

## Highlights

- This is the first multi-center randomized sham-controlled trial to evaluate the efficacy and safety of acupuncture for asymptomatic hyperuricemia with adequate power and strict adherence to the methodology of the Good Clinical Practice guidelines.
- An advantage of this trial is the use of a reasonable sham comparator, i.e., non-invasive sham acupuncture using blunt-tipped needles and following the same acupuncture process as the verum acupuncture group. This will not only promote blinding but also help to investigate the specific effect of the acupuncture treatment.
- The primary outcome of serum uric acid will be assessed by laboratory tests, which tends to increase the objectivity of the research and heighten confidence in the findings.

## Background

Asymptomatic hyperuricemia is a metabolic disease caused by purine metabolism disorder. Typically, it is defined as a serum uric acid (SUA) level greater than 6.8 mg/dL and the absence of a history of visible tophi or the onset of gout (1). Over the past few years, the prevalence of hyperuricemia and gout has increased in developed countries (2–4). This is possibly due to shifts in diet and lifestyle, such as the increased consumption of purine-rich foods, fructose, and alcoholic beverages, and the subsequent increase in obesity rates (5, 6). The prevalence of asymptomatic hyperuricemia is also high, although contemporary data are limited.

Recently, a growing body of evidence has suggested that the clinical dangers of asymptomatic hyperuricemia to human health have become increasingly prominent. It is generally believed that hyperuricemia is still the most important risk factor for the development of gout: approximately 10% of individuals with asymptomatic hyperuricemia will eventually experience a gout attack (7). The key pathological mechanism of gout is the deposition of monosodium urate (MSU) crystal (8). With the development of non-invasive methods for the identification of MSU crystals, such as ultrasound and dual-energy computed tomography (DECT), the presence of a preclinical MSU crystal deposition in asymptomatic hyperuricemia was proposed. Ultrasound evidence of MSU crystal deposition has been found in 34%–42% of patients with asymptomatic hyperuricemia (9, 10). Similarly, using DECT, investigators observed DECT urate deposits in 24% of asymptomatic hyperuricemia patients (11). Asymptomatic MSU crystal deposition may lead to both intra-articular and extra-articular damage and be a predictor for the development of other symptomatic diseases (12). Evidence from epidemiological studies of comorbidities associated with asymptomatic hyperuricemia has increased over the past 20 years, such as metabolic syndrome (13–15) and cardiovascular and renal diseases (1, 16–20). Therefore, early intervention strategies should be provided to reduce SUA among patients with asymptomatic hyperuricemia, because urate-lowering therapy (ULT) may reduce the risk of these patients developing gout or other comorbidities.

However, there is no conclusive evidence or consensus to support pharmacotherapy for patients with asymptomatic hyperuricemia. At present, ULT is not recommended for asymptomatic hyperuricemia patients in the management guidelines published by major rheumatology societies (21, 22). Only Japanese guidelines recommend ULT treatment to asymptomatic hyperuricemia patients if SUA levels are greater than 8.0 mg/dL (23). Furthermore, the benefits/harms ratio of ULT drugs for asymptomatic hyperuricemia is still unclear (24). Currently, the consensus for asymptomatic hyperuricemia management is to implement lifestyle changes to prevent the onset of hyperuricemia, such as a low-purine diet, weight loss as appropriate, and sufficient physical activity (23, 25).

Currently, attention is being paid to the effects of complementary and alternative medicine (CAM) on asymptomatic hyperuricemia patients (26). Acupuncture (also termed manual acupuncture) is an important part of CAM and is usually practiced by traditional acupuncturists. After the insertion of needles into acupoints, the manual manipulation (that is, intermittent rotation as well as lifting and thrusting) of the needles can induce de qi (a compositional sensation including soreness, numbness, distension, and heaviness), which is believed to enhance the clinical benefits of acupuncture. Previous studies have shown the benefits of acupuncture on patients with gouty arthritis (27–30). Acupuncture can promote uric acid excretion, reduce uric acid synthesis and reabsorption, and promote uric acid decomposition (31, 32). However, there have been no well-designed randomized controlled trials assessing the effects of acupuncture in adults with asymptomatic hyperuricemia. To date, only one short-term clinical trial has shown the potential benefits of acupuncture for individuals with asymptomatic hyperuricemia; however, this trial used penetrating sham acupuncture as the control, without a blinding assessment and acupuncture de qi assessment (33, 34). Hence, it is still unclear whether or not acupuncture is an effective treatment for the asymptomatic hyperuricemia population. In particular, evidence about the long-term effects of acupuncture on SUA levels in the asymptomatic hyperuricemia population is still lacking.

## Objectives

To address the lack of studies in the asymptomatic hyperuricemia population, we will investigate the efficacy and safety of acupuncture on SUA levels in adults with asymptomatic hyperuricemia, and, if found, whether or not the effect of acupuncture on SUA levels can last for at least 12 weeks. This study will be the first to use non-penetrating sham acupuncture for the control group. The de qi sensation from acupuncture will be quantified to investigate the specific effect of acupuncture on SUA levels in this clinical population.

## Methods

### Study design

This multi-center, participant-blind, parallel-group, randomized, sham-controlled clinical trial will be conducted in three hospitals in China: (1) Tongji Hospital, affiliated to Huazhong University of Science and Technology (HUST); (2) Wuhan No.1 Hospital, affiliated to HUST; and (3) Xianning central hospital, affiliated to Hubei University of Science and Technology. The protocol for this trial (version number 2.0, dated 22 June 2021) is guided by the Standard Protocol Items: Recommendations for Interventional Trials (SPIRIT 2013) and the standards for reporting clinical trials of acupuncture (35, 36) (**see** **Additional files 1** and **2**).

**Figure 1:**
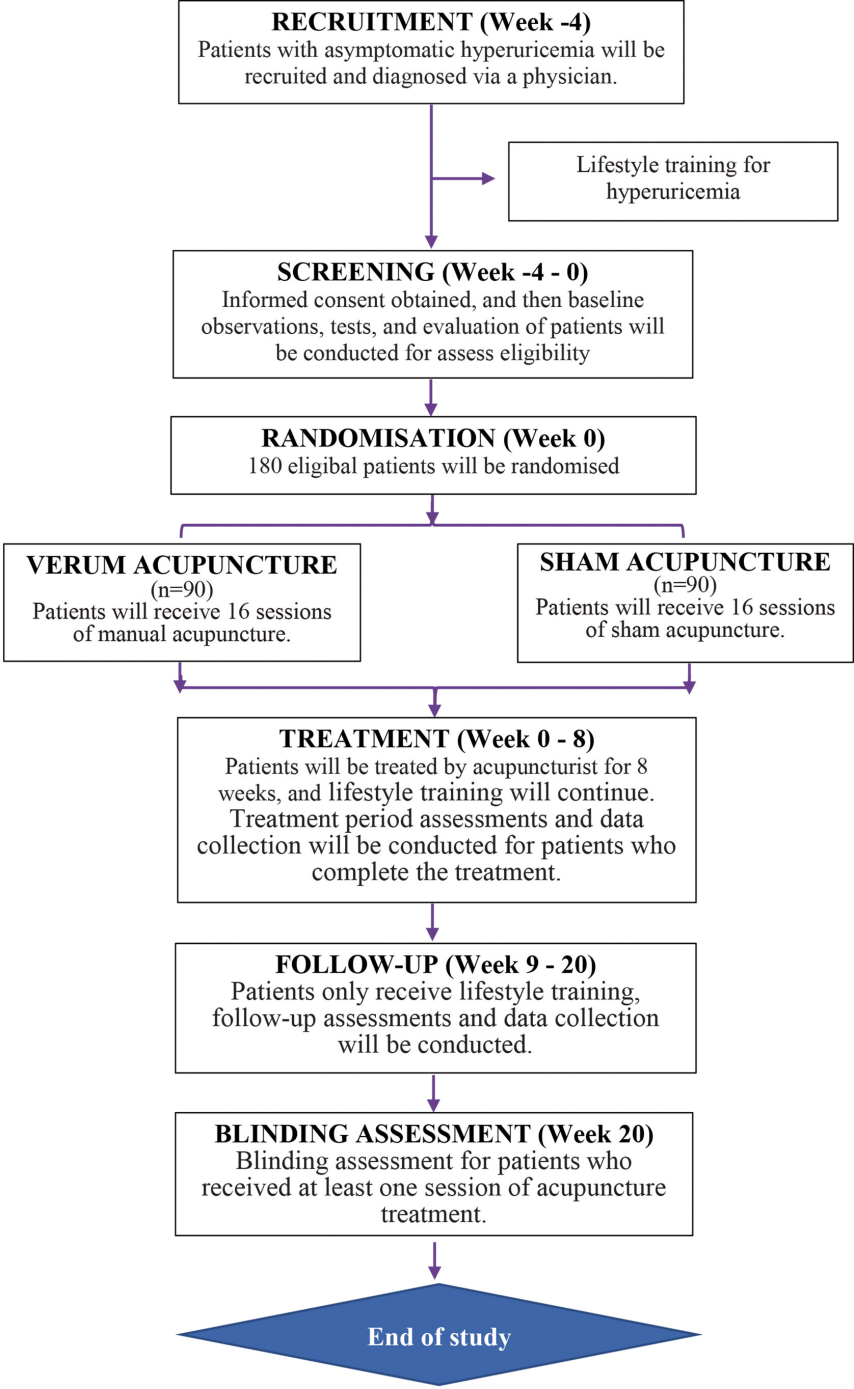
Study flow diagram.

The entire study period consists of a 4-week baseline, 8 weeks of treatment, and a 12-week follow-up. Eligible and fully informed participants will be randomly assigned to one of the following two groups in a 1:1 ratio with block randomization: the verum acupuncture group or the sham acupuncture group. All participants will provide their informed consent. In accordance with the Declaration of Helsinki, research assistants will provide detailed information about this study to participants before randomization, including the research objectives, research methods, possible benefits, and risks. Participants will be free to continue or withdraw from the trial at any time. A flow diagram of the trial is presented in **Figure 1**. **Table 1** depicts the schedule of visits and procedures in the study.

**Table 1:** Schedule of recruitment, interventions, and measures.

	Time point (weeks)						
	Baseline		Treatment		Follow-up		
	−4	0	4	8	12	16	20
Enrollment							
Screening	**○**						
Informed consent	**○**						
Previous disease history	**○**						
Medical history of HUA	**○**						
Physical examination	**○**						
Laboratory test for SUA level	**○**	**○**					
**Randomization**		**○**					
Intervention							
Verum acupuncture group		<━━━━━>					
Sham acupuncture group		<━━━━━>					
Lifestyle changes to combat HUA	<━━━━━━━━━━━━━━━━━━━━━━━━>						
Measures							
Change in SUA level			**○**	**○**	**○**	**○**	**○**
Response rate			**○**	**○**	**○**	**○**	**○**
Information on acute gouty arthritis attacks			**○**	**○**	**○**	**○**	**○**
Weight	**○**		**○**	**○**	**○**	**○**	**○**
Intake of acute medication	**○**		**○**	**○**	**○**	**○**	**○**
MASS			**○**	**○**			
AEs			**○**	**○**			
Blinding assessment							**○**

AE, adverse event; HUA, hyperuricemia; MASS, MGH Acupuncture Sensation Scale; SUA, serum uric acid.

### Recruitment

We will recruit 180 patients with asymptomatic hyperuricemia aged 18–65 years, starting from 1 June 2022. This age range was chosen to limit age-related comorbidities or other diseases. The recruitment of patients must be approved by the relevant ethics committee after the completion of clinical trial registration. Participants will be recruited in the outpatient clinics or physical examination centers of the participating hospitals through local advertising, the hospital websites. Diagnosis of asymptomatic hyperuricemia will be made by a senior physician.

### Inclusion criteria

1. Meets the diagnostic criteria for hyperuricemia, in accordance with the guidelines of the American College of Rheumatology (2012) (37).
2. Aged 18–65 years (regardless of sex),
3. SUA ≥ 7–11.0 mg/dL after 1 month of a low-purine diet,
4. No history of gouty arthritis.
5. Has not received uric acid-lowering drug treatment or stopped uric acid-lowering drug treatment ≥ 12 weeks before randomization.
6. 18.5kg/m^2^ ≤ body mass index (BMI) ≤ 30.0 kg/m^2^.
7. Written informed consent.

### Exclusion criteria

1. Secondary hyperuricemia, such as hyperuricemia induced by bone marrow and lymphoproliferative diseases, tumor chemoradiotherapy, liver cirrhosis, or drugs.
2. One of the following complications: poorly controlled hypertension [systolic blood pressure (SBP) ≥ 160 mmHg, diastolic blood pressure (DBP) ≥ 100 mmHg] or poorly controlled diabetes (hemoglobin A1c ≥ 8.4%), stroke, coronary heart disease, severe liver and kidney damage [chronic kidney disease (CKD) ≥ stage 2 or a serum creatinine, urea, alanine, or aspartate aminotransferase level more than twice the upper limit].
3. History of severe neuropsychological diseases.
4. Pregnant.
5. Illiterate or unwilling to accept acupuncture treatment.

### Randomization and blinding

The Clinical Research Center affiliated to the Tongji Hospital of HUST are responsible for the design of the randomization protocol for this study. After confirmation of eligibility, participants will be randomly allocated to either the verum acupuncture or sham acupuncture group in a 1:1 ratio. Participants will be stratified according to study center, and a permuted block randomization method with a block size of four will be used. The randomization sequences will be computer-generated using a central randomization system and according to the order of participant enrollment. Each research center will have an independent research assistant responsible for generating allocation sequences through a password-protected central randomization system. Acupuncturists will be made aware of group assignments prior to treatment using opaque, sealed envelopes.

As this is a blinded study, participants, outcome assessors, and statisticians will be blinded to treatment assignment. To maintain blinding in participants, acupuncturists will perform a standardized acupuncture ritual across both groups. Because acupuncturists perform acupuncture through the manipulation of needles, the masking of acupuncturists is difficult to achieve.

### Intervention

The intervention protocol was developed based on expert consensus. All participants will receive 16 sessions of 30-mintute verum acupuncture or sham acupuncture. They will be treated twice a week over 8 weeks. We recommend at least a 2-day interval between sessions. Acupuncturists should have obtained a license from the Ministry of Health of the People’s Republic of China and have experience in clinical practice of more than 5 years. Before performing the treatment, acupuncturists will receive centralized training, including training in acupuncture manipulation, communication skills to prevent patient loss, and the management and documentation of adverse effects from acupuncture. All participants will receive acupuncture treatment in an individual treatment room for privacy and to avoid communication between participants.

### Verum acupuncture group

**Figure 2:**
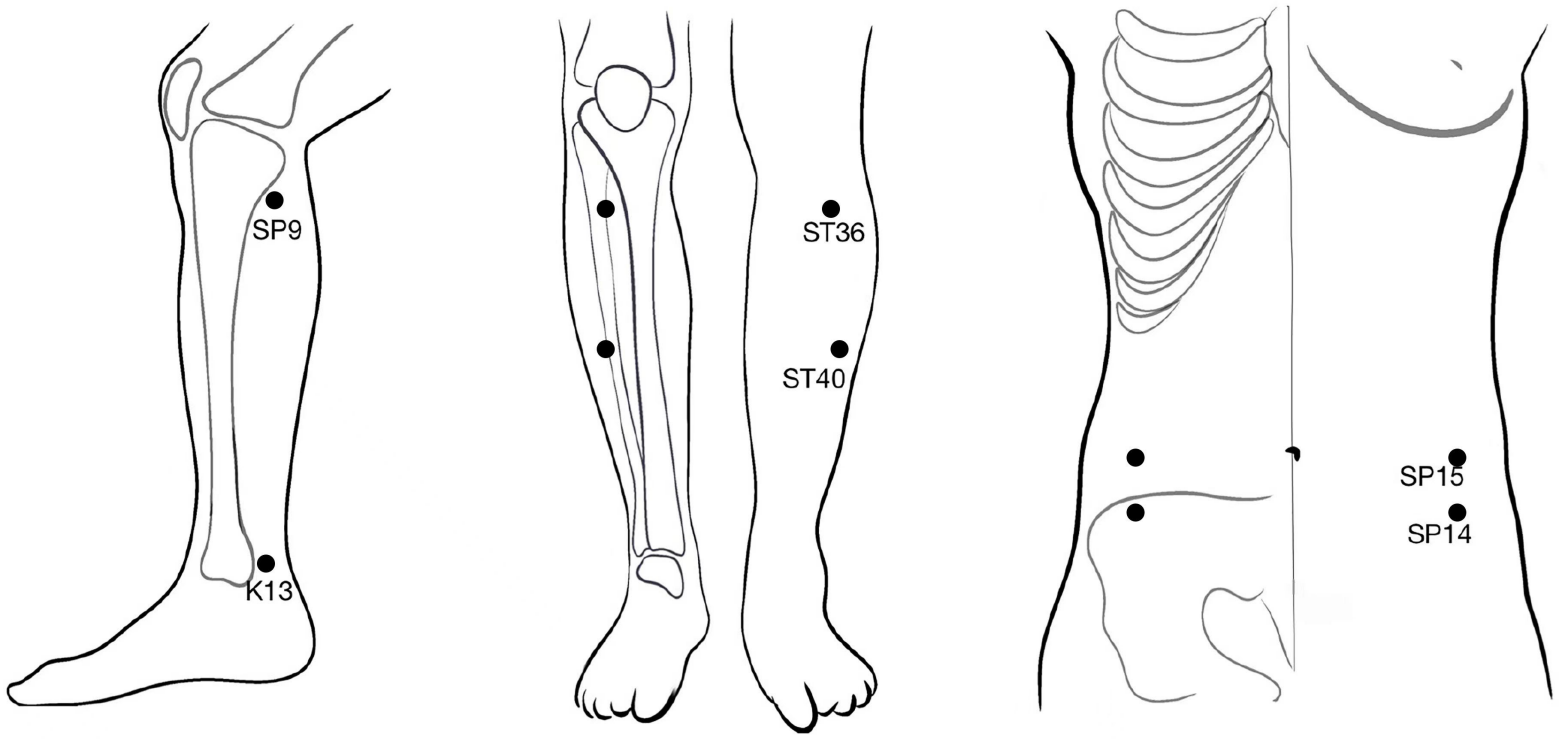
The location of used acupoints in the verum acupuncture group.

For the verum acupuncture group, penetrating needles will be used. Acupuncture will be applied at the selected acupoints, that is, Fujie (SP14), Daheng (SP15), Zusanli (ST36), Fenglong (ST40), Taixi (K13), and Yinlingquan (SP9). All acupoints are located in accordance with the WHO Standard Acupuncture Locations and are described in **Table 1** and shown in **Figure 2**. After sterilizing the local skin of the patient and the fingers of the acupuncturist with 75% ethanol, sterile disposable stainless steel acupuncture needles (0.25 mm in diameter and 40 mm in length) will be inserted into the pre-specified acupoints.

De qi, a composite sensation from acupuncture mainly comprising soreness, numbness, distension, and heaviness (38), will be induced by the manipulation of all needles (lifting and rotating 80–120 times per minute) after insertion. During the 30-minute session, the manipulation of each needle will last at least 10 s and will be repeated four times to induce lasting de qi sensations. This manipulation procedure to induce de qi has been used in our previous acupuncture trial and is believed to be an essential component of acupuncture efficacy (39).

### Sham acupuncture group

**Figure 3:**
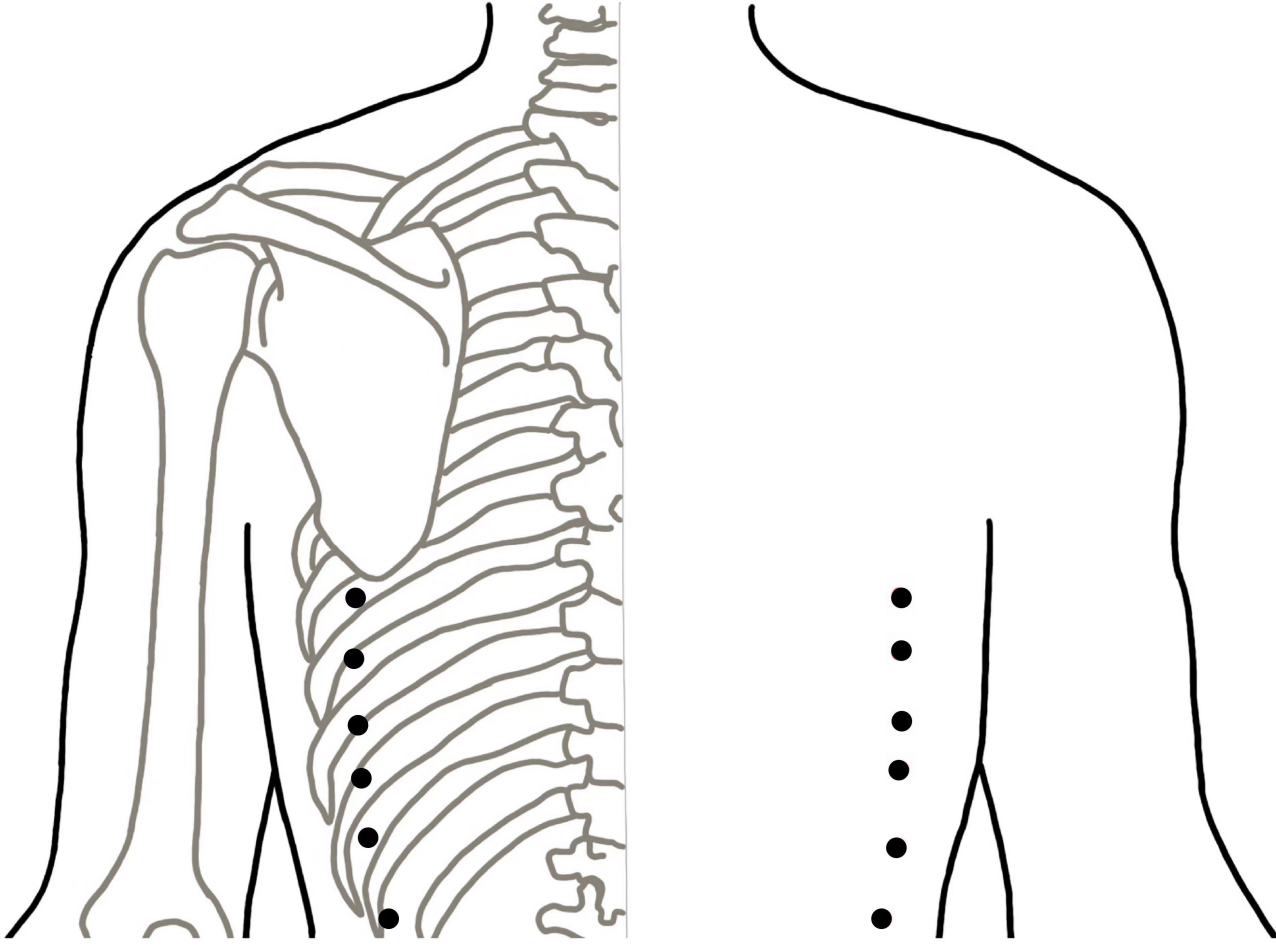
The location of used points in the sham acupuncture group.

For the sham acupuncture group, Streitberger placebo needles with blunt tips will be used (40). This is essential because penetrative sham acupuncture may still have some effect (41). Sham acupuncture will be applied at six non-meridian and non-acupoint points. The locations of the sham acupuncture points are described in **Table 2** and shown in **Figure 3**. Patients will be treated with the same number of needles and the same acupuncture rituals as the verum acupuncture group. After fixing the needles at the sham acupuncture points, acupuncturists will manipulate the needles, but avoid inducing the de qi sensation. This method of sham acupuncture has been confirmed to be effective for blinding patients in our previous acupuncture trials (39).

**Table 2:** Location of acupoints/points and manipulation techniques used for both study groups.

Acupoint/point	Location	Manipulation
Verum acupuncture group		
Fujie (SP14)	In the lower abdomen, 1.3 cun^#^ below the umbilicus and 4 cun beside the anterior midline	Inserted vertically to a depth of 1.0 cun–1.5 cun
Daheng (SP15)	In the abdomen, 4 cun lateral to the umbilicus	Inserted vertically to a depth of 1.0 cun–1.5 cun
Zusanli (ST36)	3 cun directly below Dubi,^*^ and 1 finger breadth lateral to the anterior border of the tibia	Inserted vertically to a depth of 1.5 cun–2.0 cun
Fenglong (ST40)	8 cun above the tip of the lateral malleolus, and 2 finger breadths lateral to the anterior border of the tibia	Inserted vertically to a depth of 1.5 cun–2.0 cun
Taixi (K13)	On the medial side of the foot, at the depression between the tip of the medial malleolus and the Achilles tendon	Inserted vertically to a depth of 0.5 cun–0.8 cun
Yinlingquan (SP9)	At the depression between the lower margin of the medial tibial condyle and the medial margin of the tibial condyle	Inserted vertically to a depth of 1.0 cun–1.5 cun
Sham acupuncture group		
NA 1	5 cun lateral to the 7th thoracic spine	Non-penetrating
NA 2	5 cun lateral to the 8th thoracic vertebra	Non-penetrating
NA 3	5 cun lateral to the 9th thoracic vertebra	Non-penetrating
NA 4	5 cun lateral to the 10th thoracic vertebra	Non-penetrating
NA 5	5 cun lateral to the 11th thoracic vertebra	Non-penetrating
NA 6	5 cun lateral to the 12th thoracic vertebra	Non-penetrating

#1 cun (approximately 20 mm) is defined as the width of the interphalangeal joint of the patient’s thumb.

*Dubi is in the lateral depression of the patellar ligament, when the knee is flexed.

### Lifestyle-related guidance for hyperuricemia

According to the Multidisciplinary Expert Consensus on Diagnosis and Treatment of Hyperuricemia Related Diseases in China and the 2020 American College of Rheumatology Guideline for the Management of Gout (21), general lifestyle-related recommendations for treating hyperuricemia include weight control, exercising regularly, limiting the intake of alcohol and high-purine and high-fructose foods, encouraging intake of dairy and fresh vegetables, and drinking moderate amounts of water. All participants will be asked to follow this general lifestyle advice as a basic prevention method by attending monthly lectures or studying the text provided on lifestyle guidance.

### Co-interventions

Participants will not receive any other medical treatment for asymptomatic hyperuricemia, except in an emergency. In the case of a gout attack, colchicine tablets (0.5 mg/tablet; maximal dose = 6 mg/day) will be allowed as a rescue medication.

### Strategies to improve adherence to intervention

During the screening period, we will give patients a detailed explanation of the frequency of the acupuncture treatment and the potential risks and benefits of the trial, so that participants will be fully informed before voluntarily participating in this trial. After randomization, we will focus on establishing a good doctor–patient relationship by ensuring the high quality of the treatment and the clinical environment. We will explain the purpose and necessity of each examination, treatment and review, and obtain the consent and cooperation of the patients.

### Criteria for discontinuing the allocated intervention

Acupuncture will be ended if the participant meets any of the following conditions:

1. The patient experiences a serious adverse event during or after treatment.
2. The patients is found to be pregnant after randomization.
3. The patient is found to suffer from other severe diseases after randomization that will affect their SUA level.

### Provisions for post-trial care

All of the interventions we took were harmless to the human body, so we didn't mention Post trial care.

## Outcomes

### Primary outcome

Participants’ SUA levels will be evaluated at six time points during the study: once at baseline (week 0), twice during the treatment period (4 and 8 weeks post randomization), and three times during the follow-up period (12, 16, and 20 weeks post randomization). The primary end point of this study is the reduction in SUA level from baseline to 8 weeks after randomization.

### Secondary efficacy outcomes

The reduction in SUA level at 4, 12, 16, and 20 weeks after randomization will also be evaluated. Additional secondary outcomes comprise the proportion of participants with SUA ≤ 6.0 mg/dL, the proportion of participants with SUA ≤ 7.0 mg/dL, the proportion of participants with gout attacks post randomization, body weight, and the amount of acute medication taken. These outcomes will be recorded at baseline (week 0), during the treatment period (4 and 8 weeks post randomization), and during the follow-up period (12, 16, and 20 weeks post randomization).

### Assessment of acupuncture sensation

To test the acupuncture de qi effects, all patients will be asked to complete the Massachusetts General Hospital (MGH) Acupuncture Sensation Scale (MASS), immediately following each session of acupuncture treatment.

### Blinding assessment

To test the patient-blinding effects, all patients will be asked to guess their group allocation after completing the entire course of treatment and the data collection (20 weeks post randomization). Participants will be asked “which type of acupuncture do you think you are received?” and given the following options: (A) penetrating acupuncture; (B) modified non-penetrating acupuncture; or (C) not sure.

### Assessment of safety

Adverse events (AEs) in which a causal relationship to the acupuncture cannot be ruled out will be considered an adverse reactions from the acupuncture. AEs related to acupuncture may include abnormal pain, bleeding, congestion, broken needles, bent needles, and dizziness. Any AEs will be recorded by the acupuncturists immediately following each session of acupuncture treatment.

### Baseline psychological outcome measures

To test the effects of neuropsychological factors on acupuncture, the 60-item Neuroticism-Extraversion-Openness-Five Factor Inventory (NEO-FFI) short-form test, the Acupuncture Expectancy Scale, the Beck Anxiety Inventory (BAI), the Beck Depression Inventory II (BDI- II), the Patient–Doctor Relationship Questionnaire (PDRQ-9), and the Difficult Doctor–Patient Relationship Questionnaire (DDPRQ-10) will be evaluated once at baseline.

### Reporting procedures for adverse events

In this study, the acupuncturist will assess acupuncture safety and record acupuncture-related safety data. Details of the acupuncture treatments and AEs arising from the acupuncture will be recorded in the acupuncture record form. If AEs should occur, acupuncturists will promptly take the necessary measures to ensure the patient’s wellbeing. AEs that result in any of the following events will be defined as serious: (1) fatal events, (2) life-threatening events, (3) events that require hospitalization, (4) events that result in severe impairments or disabilities of function, and (5) other clinically significant events or reactions. If any serious AEs occur, the researchers must report them to the principal investigator and the Data and Safety Monitoring Board (DSMB) within 24 hours.

### Data collection and management

#### Data collection

The investigator will conduct a baseline assessment within 1 month of obtaining informed consent. Demographic and epidemiological data will be obtained using a general information questionnaire, which will record information on age, gender, nationality, education, occupation, weight, height, previous disease history, routine physical examination, and medicine used for hyperuricemia. After receiving health education on hyperuricemia and 1 month of a low-purine diet, the serum uric acid concentrations of patients who have been enrolled in this study will be confirmed by laboratory tests.

During the treatment period, all patients will be asked to rate their acupuncture de qi sensations using the MASS immediately after the end of each acupuncture session. AEs related to acupuncture will be observed by acupuncturists. AEs, such as bent needles, stuck needles, broken needles, fainting, and infections, will be recorded. Serum uric acid concentrations will be tested at 4 and 8 weeks after randomization. During the follow-up period, serum uric acid concentrations will be tested at 12, 16, and 20 weeks after randomization. If patients have gout attacks after randomization, the time and location of the gout attack, and any medication used, will be recorded. All paper-based data will be recorded in the case report form (CRF). The data will be collected up to the time of withdrawal and will be included in the analysis unless participants specifically request for their data to be withdrawn.

#### Electronic data management

Data will be managed using the electronic data capture (EDC) system hosted at the Data Management Center of Tongji Hospital. Database builders will create electronic CRFs (eCRFs) using the CRFs and all annotations on the CRF. All collected data from the CRF will be entered into the EDC system twice by two independent investigators to ensure the accuracy of the data. After all data are checked, the database will be locked. Any modifications after the database is locked will be discussed by the principal investigator and sighed by the data entrant. Both the paper and electronic documents will be held for at least 5 years after publication.

#### Monitoring and quality control

The trial protocol was formulated by experts in the fields of acupuncture, rheumatology, statistics, and public health. When a revision is needed, an amended protocol will be resubmitted to the Clinical Trial Ethics Committee of Tongji Medical College, HUST, for review. To guarantee the quality of the study, we will provide a standardized training course to all research centers before recruitment, which includes the methods for screening patients, acupuncture delivery, data collection, data input, and an introduction to the database. After initiation, the trial will be monitored regularly by inspectors from the Tongji Hospital, Tongji Medical College, HUST. A Data and Safety Monitoring Board (DSMB) has been set up to monitor the performance and safety of the trial, which is composed of five experts from different fields based on the Chinese mainland. Any modifications to the data can be traced in the Beijing Absolute Clinical Data System electronic data capture (AbsEDC) platform. The reasons and details for drop-outs or withdrawals will be documented.

#### Sample size

The primary efficacy end point of this trial is the reduction in SUA from baseline at 8 weeks after randomization. The sample size of this study is driven by the superiority test of the primary efficacy endpoint, aiming to demonstrate a greater reduction in SUA concentration in the verum acupuncture group compared to the sham acupuncture group. Based on the pilot study and a previous clinical trial (33), we anticipate that SUA concentration will be reduced by an average of 0.58 mg/dL ± 1.15 mg/dL more in the verum acupuncture group than in the sham acupuncture group. Therefore, a sample size of 142 participants (71 patients in each group) is required to detect the difference between study groups with 85% power at an alpha level of 0.025 (one-sided). Considering an expected attrition rate of 20%, we plan to enroll at least 180 participants into this study.

#### Statistical analysis

Statistical analyses will be performed using SAS (SAS Institute, v.9.4) by an independent statistician who is blinded to group allocation. Efficacy analyses will be performed primarily on the full analysis set (FAS). A sensitivity analysis will be performed on the per-protocol set (PPS) if deemed necessary.

The primary end point will be summarized descriptively, and the hypothesis will be tested based on the analysis of covariance (ANCOVA) model with the intervention group as the main effect and baseline SUA and study center as covariables.

For other end points, continuous data yielding normal distributions will be summarized using means and standard deviations. Differences between the two groups will be tested using Student’s *t*-test. For results with non-normal distributions, data will be expressed using medians and quartiles, and comparisons between the two groups will be performed using the Mann–Whitney *U* test. Categorical end points will be summarized in terms of frequencies and percentages, and comparisons between the two groups will be performed using the Pearson *χ*² test or Fisher’s exact test, as appropriate. The statistical test for the primary end point will be at the one-sided 0.025 alpha level. Unless otherwise specified, all other end points will be tested at the two-sided 0.05 alpha level.

#### Patient and public involvement

Patients will not be involved in the design, conduct, or reporting of this study. The results of the study will be disseminated via a research article in a peer-reviewed journal. The results will also be disseminated at scientific meetings. Patients will also be informed of the results by follow-up staff at the research centers once the trial has finished.

## Discussion

Historically, hyperuricemia was considered of little clinical importance in the absence of gout or kidney stones. Yet over the past 20 years, increasing evidence has suggested that sustained elevation of serum urate levels can result in the supersaturation of body tissues with urate, leading to the deposition of sodium urate crystals in and around joints (1, 42, 43). As such, patients with hyperuricemia who have not yet developed gout symptoms may also experience joint damage. Therefore, early effective therapy is the key to preventing the onset of joint damage and other complications. However, whether or not, and when, conventional ULT drugs should be recommended to patients with asymptomatic hyperuricemia is still controversial. Some patients do not respond well to urate-lowering drugs, or cannot tolerate the adverse effects of the drugs. These limitations can lead to low adherence and disease aggravation. In this prospective trial, we aim to evaluate the efficacy and safety of acupuncture for the treatment of asymptomatic hyperuricemia by analyzing SUA outcomes.

This trial was designed to meet the methodological requirements of adequate power, allocation concealment, and necessary blinding, as outlined by the Good Clinical Practice guidelines (44). In the verum acupuncture group, manual acupuncture will be performed by licensed traditional Chinese acupuncturists. This is a form of traditional acupuncture therapy that has been used to treat various diseases in China and has been practiced for more than 2,000 years. After acupuncture needles are inserted into specific acupoints, standard manual manipulation of the needles generates de qi sensations, which play a key role in the specific effect of acupuncture (45). We will evaluate the dynamic changes in SUA levels of 180 eligible participants over a relatively long follow-up period (12 weeks). If acupuncture can reduce patients’ SUA levels to an optimal range and its clinical benefits are still reported at least 12 weeks after treatment is ended, this will compensate for the low persistence of effects from conventional urate-lowering therapy.

In acupuncture clinical studies, blinding and control treatments are always a challenge. An appropriate placebo acupuncture should be both physiologically ineffective and indistinguishable from true acupuncture. To observe the specific effects of acupuncture on asymptomatic hyperuricemia, we will use non-invasive placebo needles in the sham acupuncture group and the same acupuncture ritual will be performed in both groups, as described in our previous study (39).

It is accepted that uric acid can be affected not only by lifestyle factors (such as a high-purine diet, high alcohol intake, and a lack of exercise), but also by obesity, diabetes, comorbidities, and medications (46). Thus, it is crucial to consider the influence of these confounding factors when conducting acupuncture trials on an asymptomatic hyperuricemia population. In order to control the influence of lifestyle on uric acid, we will provide lifestyle-related guidance for hyperuricemia to all participants from baseline to the end of the follow-up period. All participants will receive the same recommendations using standardized written lifestyle guidance or by attending a monthly lecture. For improved adherence to the lifestyle prescription, all participants will be requested to record a lifestyle-related questionnaire and submit it to research assistants every 4 weeks (**see** **Additional file 3**). Moreover, we also considered the influence of age, obesity, comorbidities, and medications on uric acid through strict inclusion and exclusion criteria.

The study does, however, have limitations. First, acupuncturists cannot be blinded, owing to the nature of acupuncture. As such, we cannot completely avoid an influence on study outcomes from the subjective behavior of the acupuncturists. Acupuncturists will perform the same treatment procedure in both groups, which may partially compensate for the lack of blinding in the acupuncturists. Second, sex differences are not included in the randomization adjustment factors, although evidence suggests that sex can influence serum uric acid levels (46). Third, the duration of this study may not be long enough to demonstrate a long-lasting effect from acupuncture, although we will observe whether the treatment effect from acupuncture can persist until 12 weeks after treatment.

At the time of submission of this protocol, participant enrollment is progressing well and data collection is well underway. Participant recruitment started on 13 July 2022 and is expected to end on 30 December 2024. We expect this study will provide reliable evidence regarding the efficacy and safety of acupuncture for asymptomatic hyperuricemia. If so, acupuncture can be recommended as an effective alternative treatment for patients with asymptomatic hyperuricemia when lifestyle changes have failed or patients meet the contraindications for urate-lowering drugs.

## Confidentiality

All information collected from participants will be stored securely. All paper information will be stored in locked filing cabinets in areas with limited access. All the information entered into an electronic database will be secured with password-protected access systems.

## Patient and public involvement

Neither patients nor the public have been involved in the Patients will not be involved in the design, conduct, or reporting of this study. The results of the study will be disseminated in via a research article at in a peer-reviewed journal. The results will also be disseminated at scientific meetings. Patients will also be informed of the results by follow-up staff at the research centers, once the trial has finished.

## Ethics statement

The protocol (version number: 2.0 dated 15 June 2021) for this trial has been approved by the Clinical Trial Ethics Committee of Tongji Medical College, HUST (No. 2021-S135). It was registered on 31 May 2022 in the Clinical Trials Registry (No. NCT05406830). Written informed consent will be obtained before randomization.

## Author contributions

L-LY, S-HT, ZC, and YW contributed to the design of this study. L-LY and C-NL drafted the first draft of this manuscript. S-HT obtained the funding. M-YF contributed to the writing of this article. W-HL contributed to the sample size calculation and draft statistical plan. C-HZ and W-XX performed the acupuncture treatment. BW, F-PL, and YH carried out the participant randomization process. All authors revised the manuscript critically and approved the submission.
