# Supplementary material for: Evaluating the effectiveness and safety of acupuncture on serum uric acid in asymptomatic hyperuricemia population: a randomized controlled clinical trial study protocol
Source: Front Endocrinol (Lausanne). 2023 Oct 13;14:1218546. doi: 10.3389/fendo.2023.1218546 (PMC10611493; doi:10.3389/fendo.2023.1218546)
Supplement: Supplementary file 3 [file Table_3.docx]

**Evaluate date：**□□□□(year)□□(month)□□(day)

**Dieting management**

**Please fill in all blanks according to your actual situation in eating and moving. And please fill out with √when it fit with your situation, if not, you can keep it blank.**

|  | **Monday** | **Tuesday** | **Wednesday** | **Thursday** | **Friday** | **Saturday** | **Sunday** |
| --- | --- | --- | --- | --- | --- | --- | --- |
| **The diet need to avoid or limit** | | | | | | | |
| 1 Eat offal such as liver, brain and kidney |  |  |  |  |  |  |  |
| 2 Drink wine especially beer and liquor |  |  |  |  |  |  |  |
| 3 Eat seafood |  |  |  |  |  |  |  |
| 4 Eat dense broth and gravy |  |  |  |  |  |  |  |
| 5 Eat red meat like beef,mutton and pork |  |  |  |  |  |  |  |
| 6 Eat dish |  |  |  |  |  |  |  |
| 7 Eat food with high-fat, such as deep-fry food, skin of animals and so on |  |  |  |  |  |  |  |
| 8 Drink sugary beverage like cola, orange juice and apple juice |  |  |  |  |  |  |  |
| 9 Eat the food with abundant sugar(for example: persimmon, banana, wax myrtle, honey, pomegranate, orange, fig, caster sugar, brown sugar, flour, sugar cane, yam, beet and so on) |  |  |  |  |  |  |  |
| **Encourage to be ate** | | | | | | | |
| *10 Drink water per day≥2000ml，about 4 bottles of mineral water |  |  |  |  |  |  |  |
| *11 Eat the fresh fruit and vegetables have abundant potassium and vitamins C（500g/d）, like bananas, pears, peaches, pineapples, kiwi fruits and cherries. |  |  |  |  |  |  |  |
| *12 Drink skimmed or low-fat milk or yoghurt（300ml/d） |  |  |  |  |  |  |  |
| *13 Eat low-glycemic-index cereals (roughage, pulses) |  |  |  |  |  |  |  |
| **Behavior** | | | | | | | |
| 14 Smoke |  |  |  |  |  |  |  |
| 15 Stay up, go asleep later than 11p.m., slumber in night less than 7 hours. |  |  |  |  |  |  |  |
| *16 Keep aerobics about 20 minutes each time, such as running, swimming, skipping and ball-sports |  |  |  |  |  |  |  |
